# The Problem of Rural Midwifery

**Published:** 1907-10-26

**Authors:** 


### Nursing Administration.

### THE PROBLEM OF RURAL MIDWIFERY.

(*Concluded from page* 74.)

In a previous article it was pointed out that the districts which will be affected by the disappearance of the amateur midwife in 1910 are precisely those in which it is idle to expect that professional midwives will be found. Nobody is going at this hour to regret the effacement of the "wise women." Their ignorance is only equalled by their self conceit; they inflict a vast amount of unnecessary torture on their patients and a vast amount of ill-health is without doubt also entailed on those introduced into the world by these worthy people, whose services we are still at a loss to supply. The problem is rendered all the more difficult of solution by the fact that the "*bona fide* midwife," whose right to practise is not disturbed in 1910, is also rapidly dying out. Few people realise to how large an extent the practice of midwifery in this country until some few years ago was confined to ancient women. We have before us a report on the number of midwives practising in Oxfordshire. At the time of the passing of the Act it was ascertained that at least 204 midwives were pursuing their calling in this county. In all probability the number actually at work was much larger, since in many instances no returns were received from those likely to be acquainted with the facts. The number of certified midwives in the county is now 84, and a general impression prevails among women who are not on the roll that they are liable to conviction if they practise without being certified. Out of these 84 midwives no fewer than 26 are over 60 years of age, and some are over 70. Thus the number of midwives which was reduced by more than half at the passing of the Act, is likely, through superannuation at no very distant date, to be reduced again by about one-fourth. Is it not clear that immediate and universal action can alone save the mothers in scattered districts from the direst distress? Either they must be driven to have recourse to the workhouse, and thus add one more cogent force to those which are driving the labourer off the land, or their needs must be considered, not spasmodically in regions where there happens to be someone with time and energy to raise funds for supporting a nurse, but as part of a well-thought-out plan which can be adopted without difficulty in every rural district throughout the country. It is undoubtedly on the Guardians of the poor that the burden must ultimately fall of making due provision for such mothers as are unable to procure for themselves aid in child-birth such as they can afford to pay for. We may sympathise with the malodour which attaches to the relief meted out in rural England through the chilly medium of the rates, but it is well to recognise at once that through the rates alone can the necessary machinery be procured which is to render the situation tolerable. Do we not all know village after village in which there is no public spirit, no money, no organising power? To expect that such places can support a voluntary nurse is to pave the way for inevitable disaster. The nurse who has come to be a necessity in every Union can only in by far the greater majority of Unions be supported by a public body, and since the Parish Councils have no powers to levy rates for such a purpose, it falls to the Guardians of the Poor to see that the nurse is provided. There are two weighty reasons in favour of this duty being performed by the Guardians when not undertaken voluntarily. The first is a reason of finance. The parochial nurse would not merely alleviate much distress, but would in all probability repay her salary to the ratepayers by her effect in keeping people off the rates. In the sick wards of many rural Unions are to be found a number of chronic invalids who are in residence mainly on account of the impossibility of their being cared for in their own homes. A host of ulcerated legs, of bad bed-sores, and of senile cases could be perfectly well cared for in their own homes were there a nurse available to perform the dressings, attend to the comfort of the patients, and give those simple instructions as to food and cleanliness, for want of which their condition at home becomes unbearable. The experience of all district nurses shows the extent to which district nursing has acted in reducing the number of inmates in the workhouse, and for this reason the Guardians in many places have cordially voted subsidies towards the upkeep of the nurse. From this to assuming the full burden of maintaining the nurse is but a step. In view of the expense attendant on the maintenance of properly equipped lying-in wards such as would be required if not merely occasional cases of destitution were to be received, but if all whose wages were below a certain average were driven by necessity to resort to them, we believe the parochial nurse will shortly be recognised as indispensable. We believe, moreover, that, independently of financial reasons, the association of the parochial nurse with the hated name of parish relief will have a far-reaching effect for good. Has not the time gone by when it was thought necessary that the amounts contributed towards the relief of the poor should be associated with hate, rather than love? For hundreds of years we have acted the part of the good Samaritan to the necessitous in our midst, and carried him to so rude an inn that he has craved rather to be suffered to die in peace by the road-side than to submit to our interference. Have we succeeded by all this calculated animosity on the part of Poor-law administration in eradicating the taint of pauperism from our midst? We believe that the nurse has come to show the student of political economy "the more excellent way." We believe that through the provision of merciful aid in sickness, and in the pangs of childbirth, the hour may be approaching when the poor shall no longer curse the law which provides for their necessities, but shall receive with gratitude a gift offered in the spirit of love, and shall learn from the trained intelligence which soothes their distress how to rear their children in health and independence.

